# A Human Periodontal Ligament Fibroblast Cell Line as a New Model to Study Periodontal Stress

**DOI:** 10.3390/ijms21217961

**Published:** 2020-10-27

**Authors:** Matthias Weider, Agnes Schröder, Denitsa Docheva, Gabriele Rodrian, Isabel Enderle, Corinna Lesley Seidel, Darja Andreev, Michael Wegner, Aline Bozec, James Deschner, Christian Kirschneck, Peter Proff, Lina Gölz

**Affiliations:** 1 Department of Orthodontics and Orofacial Orthopedics, University Hospital of Erlangen, Friedrich-Alexander University Erlangen-Nuernberg, Glueckstr. 11, 91054 Erlangen, Germany; gabriele.rodrian@uk-erlangen.de (G.R.); isabel.enderle@uk-erlangen.de (I.E.); corinna.boeck@uk-erlangen.de (C.L.S.); lina.goelz@uk-erlangen.de (L.G.); 2 Department of Orthodontics, University Hospital Regensburg, Franz Josef Strauss Allee 11, 93053 Regensburg, Germany; agnes.schroeder@ukr.de (A.S.); christian.kirschneck@ukr.de (C.K.); peter.proff@ukr.de (P.P.); 3 Experimental Trauma Surgery, Department of Trauma Surgery, University Hospital Regensburg, Franz Josef Strauss Allee 11, 93053 Regensburg, Germany; denitsa.docheva@ukr.de; 4 Department of Medicine 3, Rheumatology and Immunology, University of Erlangen-Nuremberg, Glueckstr. 6, 91054 Erlangen, Germany; darja.andreev@uk-erlangen.de (D.A.); aline.bozec@uk-erlangen.de (A.B.); 5 Institut für Biochemie, Emil-Fischer-Zentrum, Friedrich-Alexander-Universität Erlangen-Nürnberg, Fahrstr. 17, 91054 Erlangen, Germany; michael.wegner@fau.de; 6 Department of Periodontology and Operative Dentistry, University Medical Center of the Johannes Gutenberg University, 55131 Mainz, Germany; james.deschner@uni-mainz.de

**Keywords:** mechanical loading, orthodontic tooth movement, bone remodeling, cell culture techniques

## Abstract

The periodontal ligament (PDL) is exposed to different kinds of mechanical stresses such as bite force or orthodontic tooth movement. A simple and efficient model to study molecular responses to mechanical stress is the application of compressive force onto primary human periodontal ligament fibroblasts via glass disks. Yet, this model suffers from the need for primary cells from human donors which have a limited proliferative capacity. Here we show that an immortalized cell line, PDL-hTERT, derived from primary human periodontal ligament fibroblasts exhibits characteristic responses to glass disk-mediated compressive force resembling those of primary cells. These responses include induction and secretion of pro-inflammatory markers, changes in expression of extracellular matrix-reorganizing genes and induction of genes related to angiogenesis, osteoblastogenesis and osteoclastogenesis. The fact that PDL-hTERT cells can easily be transfected broadens their usefulness, as molecular gain- and loss-of-function studies become feasible.

## 1. Introduction

The periodontal ligament (PDL) is a thin sheet-like structure surrounding teeth, serving to anchor teeth in alveolar bones. It consists of different kinds of connective tissue fibers. This tissue has a high capacity of proliferation and of remodeling, enabling a constant width, despite different kinds of mechanical stress such as occlusal pressure and orthodontic force [1,2]. PDL fibroblasts are the main cells surrounding teeth, besides osteoblastic lineages constituting the bones. These fibroblasts fulfill a rich repertoire of functions such as tissue homeostasis and the establishment of a collagenous extracellular matrix (ECM) by secreting structural proteins, but also regulatory functions in defense reactions of the innate immune system [1,2]. PDL fibroblasts also play a major role in sensing and mediating mechanical load during occlusion of teeth in development and during orthodontic tooth movement (OTM) [3,4]. They sense compressive or tensile forces and trigger pro-inflammatory responses. One of the first steps within this process of mechanotransduction is the induction of prostaglandin-endoperoxide synthase 2 (*PTGS2*, also called cyclooxygenase 2), which catalyzes the synthesis of the eicosanoid prostaglandin H2 from arachidonic acid [5,6]. Prostaglandin H2 is then converted to different eicosanoids such as prostaglandin E2 (PGE2) by other enzymes. These eicosanoids fulfill functions in diverse physiological reactions such as inflammation and other immune responses, regulation of cell growth and of blood pressure [5,6].

To enable OTM, alveolar bone has to be resorbed by osteoclasts. Cytokines controlling the proliferation and differentiation of osteoclast precursors are macrophage colony stimulatory factor (M-CSF) and receptor activator of nuclear factor kappa-Β ligand (RANK-L), both of which are induced during OTM [7,8,9,10,11]. Thus, OTM is enabled by a pseudo-inflammatory reaction with bone resorption as the rate-limiting step [12]. Besides bone resorption, an extensive re-organization of the ECM also takes place during OTM [13,14]. This includes degradation of old ECM by matrix metalloproteases [15] as well as deposition of new collagen fibrils [14]. Other factors induced during OTM include vascular endothelial growth factor A (VEGF-A), a mitogen promoting angiogenesis and vasculogenesis [7] and alkaline phosphatase (ALPL), which plays a role in bone mineralization [16].

A well-established model to study mechanical stress on PDL (as it happens in the early phases of OTM) under in vitro conditions is the application of compressive force onto human periodontal ligament fibroblasts (hPDLF) [16,17,18,19,20,21,22]. This relies on the availability of primary hPDLF, demanding a constant supply with fresh cells from human donors. To facilitate research on OTM or mechanical stress on PDL fibroblasts in general, we set out to study the applicability of a cell line generated from hPDLF by lentiviral gene transfer of human telomerase reverse transcriptase (hTERT) resulting in stable transgene expression and cell immortalization [23]. We hypothesized that this cell line would respond to compressive force in a similar way as primary hPDLF and tested our hypothesis with established protocols. 

## 2. Results

**Figure 1:**
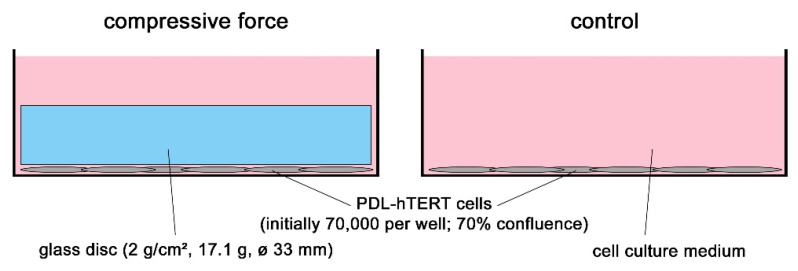
In vitro model for compressive mechanical strain on PDL-hTERT cells. Compressive force is applied by sterile glass disks (17.1 g, 2 g/cm^2^) onto PDL-hTERT cells growing adherently in 6 well cell culture plates under standard cell culture conditions in rich medium. Cells without glass disks serve as controls. Cells are incubated for different time spans and the amounts of analytes are referenced to the start of experiments (T = 0 h) and to each respective control (T = 6 h, 24 h, 48 h and 72 h).

To investigate the response of the PDL-hTERT cell line to mechanical stress, we applied compressive force simulating early phases of orthodontic treatment in vitro according to well-established protocols [3,16,19,24]. Compressive force was applied with glass disks (Figure 1) for 6 h, 24 h, 48 h and 72 h referring to previous studies [3,16,19,25]. 

### 2.1. Kinetics of Pro-Inflammatory Markers in PDL-hTERT Cells

**Figure 2:**
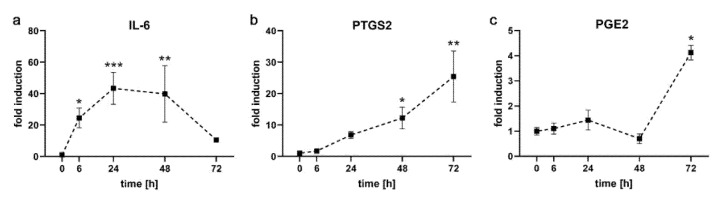
Kinetics of pro-inflammatory markers in PDL-hTERT cells under compressive load. Expression of pro-inflammatory genes *IL-6* (**a**) and *PTGS2* (**b**) at the specified time points was determined via qRT-PCR, amounts of PGE2 in cell culture medium were measured by ELISA (**c**). Values were referenced to arithmetic means at T = 0 h and are shown as fold over non-treated controls at the respective time points ±SEM. *n* = 2, *n* = 6, * 0.01 ≤ *p* < 0.05, ** 0.001 ≤ *p* < 0.01, *** 0.0001 ≤ *p* < 0.01.

Primary hPDLF respond to compressive force with an up-regulation of pro-inflammatory markers [16,25]. We therefore analyzed the expression of the pro-inflammatory genes interleukin-6 (*IL-6*) and prostaglandin-endoperoxide synthase 2 (*PTGS2*) in the PDL-hTERT cell line and the secretion of prostaglandin E-2 (PGE2) into cell culture medium upon exerting compressive force (Figure 2). As expected, we found a significant increase of these markers during the time course of our experiments. For *IL-6*, we already detected a statistically significant strong induction after 6 h of compressive load (24.53 ± 6.33-fold over T = 0 h). The strongest induction was observed at 24 h (43.35 ± 10.19), followed closely by 48 h (39.84 ± 17.94). At the last time point analyzed, expression of IL-6 had dropped to levels close to the start of the experiment (10.56 ± 1.13). 

Expression of *PTGS2* followed a slower time course. Induction was not seen until 24 h (6.82 ± 1.06) and continuously rose (12.23 ± 3.45 at 24 h) until it reached its statistically significant maximum at 72 h (25.46 ± 8.14). Accordingly, levels of secreted PGE2 did not reach statistically significant induction until 72 h (1.11 ± 0.23 at 6 h, 1.45 ± 0.40 at 24 h, 0.70 ± 0.19 at 48 h and 4.13 ± 0.30 at 72 h).

### 2.2. Expression Kinetics of Genes Involved in Formation of Extracellular Matrix in PDL-hTERT Cells

**Figure 3:**
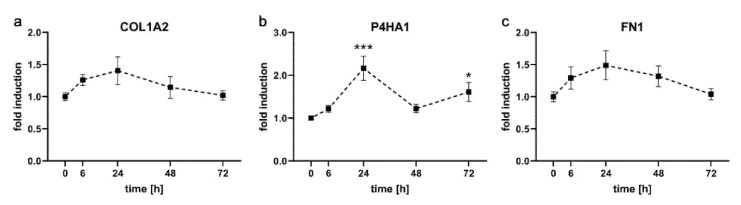
Expression kinetics of genes involved in formation of extracellular matrix in PDL-hTERT cells under compressive load. Expression of *COL1A2* (**a**), *P4HA1* (**b**) and *FN1* (**c**) at the specified time points was determined via qRT-PCR, values were referenced to arithmetic means at T = 0 h and are shown as fold over non-treated controls at the respective time points ±SEM. *n* = 2, *n* = 6, * 0.01 ≤ *p* < 0.05, *** 0.0001 ≤ *p* < 0.01.

We then investigated the expression of genes involved in formation of extracellular matrix (ECM) because they are also known to be influenced in primary hPDLF by compressive force [16,25]. As representative genes, we chose Collagen alpha-2(I) chain (*COL2A1*), Prolyl 4-hydroxylase subunit alpha-1 (*P4HA1*) and Fibronectin (*FN1*) for analysis by qRT-PCR (Figure 3). All three genes showed a moderate induction during the time course of treatment, followed by decreases down to starting point levels (*COL1A2*: 1.26 ± 0.09, 1.41 ± 0.22, 1.14 ± 0.22 and 1.02 ± 0.07, *P4HA1*: 1.22 ± 0.08, 2.17 ± 0.28, 1.22 ± 0.10 and 1.61 ± 0.22, *FN1*: 1.29 ± 0.17, 1.49 ± 0.23, 1.32 ± 0.16 and 1.04 ± 0.09 at 6 h, 24 h, 48 h and 72 h, respectively). Only the expression of *P4HA1* was significantly up-regulated after 24 h. 

### 2.3. Expression Kinetics of Genes Involved in Angiogenesis, Osteoblastogenesis and Osteoclastogenesis

**Figure 4:**
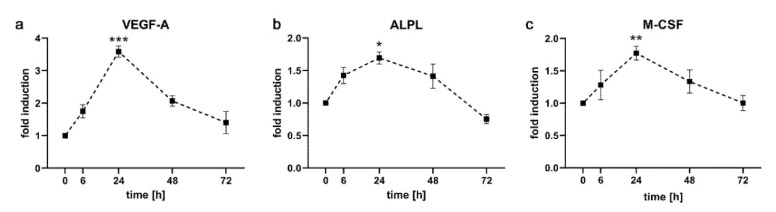
Expression kinetics of genes involved in angiogenesis, osteoblastogenesis and osteoclastogenesis in PDL-hTERT cells under compressive load. Expression of *VEGF-A*, involved in formation of blood vessels (**a**), *ALPL*, involved in formation of osteoblasts (**b**) and *M-CSF*, involved in differentiation of osteoclasts (**c**) at the specified time points was determined via qRT-PCR. Values were referenced to arithmetic means at T = 0 h and are shown as fold over non-treated controls at the respective time points ±SEM. *n* = 2, *n* = 6, * 0.01 ≤ *p* < 0.05, ** 0.001 ≤ *p* < 0.01, *** 0.0001 ≤ *p* < 0.01.

Vascular endothelial growth factor A (VEGF-A) is a signaling molecule, which is essential for formation of blood vessels and is induced by compressive force in hPDLF [16,25]. Expression of *VEGF-A* in PDL-hTERT cells started with a weak induction at 6 h (1.75 ± 0.21), was robustly induced after 24 h (3.59 ± 0.17) and dropped again to lower levels at later time points (2.07 ± 0.16 at 48 h and 1.40 ± 0.34 at 72 h; Figure 4a).

Alkaline phosphatase (ALPL) plays a key role in skeletal mineralization and is also up-regulated during application of compressive force to hPDLF [16,25]. We could show a weak but statistically significant induction of *ALPL* in early phases of compressive force on PDL-hTERT cells (1.43 ± 0.12 at 6 h, 1.69 ± 0.09 at 24 h), which ceased again at later time points (1.41 ± 0.19 at 48 h, 0.75 ± 0.07 at 72 h; Figure 4b).

M-CSF is one of the critical factors for activation of osteoclasts and its level in gingival crevicular fluid increases during OTM [7]. We could recapitulate this increase in our in vitro experiments, showing a weak induction at 6 h (1.28 ± 0.23) that reached a statistically significant maximum at 24 h (1.77 ± 0.11) and decreased again to starting levels (1.34 ± 0.18 at 48 h and 1.00 ± 0.12 at 72 h; Figure 4c). 

### 2.4. Transfection Efficiency of PDL-hTERT Cells

**Figure 5:**
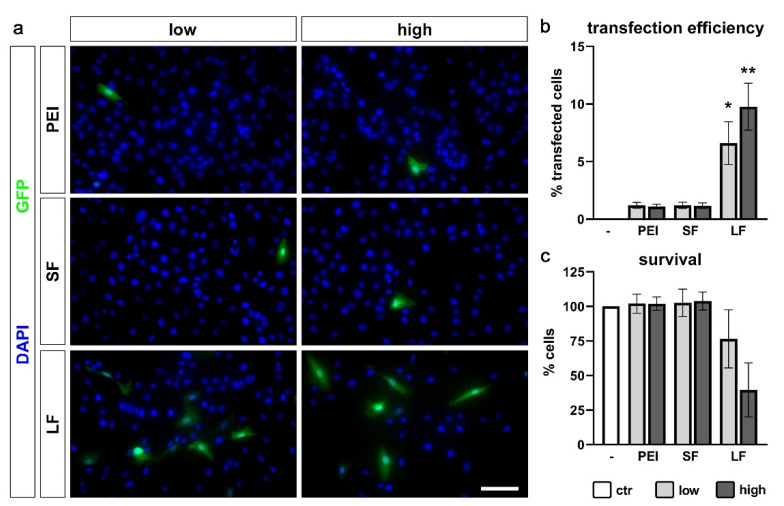
Transfection efficiency of PDL-hTERT cells. Cells were transfected with PEI, SuperFect (SF) and Lipofectamine 2000 (LF) in low and high amounts. (**a**) Representative images of GFP-positive transfected cells (green) and DAPI-stained nuclei of all cells (blue). Scale bar: 100 µm. (**b**) Quantification of transfection efficiency. GFP-positive transfected cells per field of view were set in relation to the number of DAPI-positive cell nuclei per field of view and are presented as % ±SEM. Untransfected cells served as control (ctr). (**c**) Cell viability was calculated by counting DAPI-positive nuclei per field of view and set in relation to DAPI-positive nuclei per field of view of untransfected cells (ctr) and are presented as % ±SEM. *n* = 3 biological replicates performed as triplicates with three analyzed photographs per singlet. * 0.01 ≤ *p* < 0.05, ** 0.001 ≤ *p* < 0.01.

A high transfection efficiency would allow a broad application of techniques for the PDL-hTERT cell line. We therefore tested transfection efficiency of PDL-hTERT cells with three different transfection reagents: Polyethylenimine (PEI), SuperFect and Lipofectamine 2000. Each reagent was used in two different concentrations. PEI and SuperFect yielded only minor transfection efficiencies (1.20% ± 0.25% and 1.10% ± 0.20% with low and high amounts of PEI, and 1.20% ± 0.26% and 1.17% ± 0.23% with low and high amounts of SuperFect, respectively; Figure 5a,b). However, Lipofectamine 2000 in low amounts delivered reasonable transfection efficiency (6.60% ± 1.86%; Figure 5a,b) with high cell viability (76.5% ± 21.06% of untransfected control cells; Figure 5c). Increased amounts of Lipofectamine 2000 yielded even higher transfection efficiencies (9.77% ± 2.04%) at the cost of cell viability (39.60% ± 19.57% of untransfected control cells; Figure 5a–c).

## 3. Discussion

In our in vitro study, we tested the potential of the immortalized human PDL-hTERT cell line to analyze effects of mechanical stress in the periodontium. Therefore, we followed well-established protocols of physiological compressive force of 2 g/cm^2^ via glass disks that use primary hPDLF [16,17,18,19,20,21]. We expected similar responses of the PDL-hTERT cell line to compressive strain as of primary cells. When we investigated pro-inflammatory markers, we could see induction of the prostaglandin-endoperoxide synthase *PTGS2* and increased secretion of the analyzed downstream product PGE2 into the medium. This is in line with previous in vitro findings using primary hPDLF [3,16,26] and with the discovery of increased PGE2 levels in gingival crevicular fluid of human patients in early phases (24 h and 48 h) of OTM [27,28]. Moreover, we detected a strong, temporary induction of *IL-6* gene expression that is also a characteristic response of primary hPDLF to mechanical load [16,25,26,29].

Changes in the expression of ECM-related genes *COL1A2*, *P4HA1* and *FN1* were similar to the ones in primary hPDLF [16], with the exception that *COL2A1* induction did not reach significance. This induction of ECM-reorganizing genes is also in accordance to animal models, where an increase in newly deposited collagen fibrils was seen after seven days of OTM [14]. 

We noticed an increased expression of angiogenesis-inducing *VEGF-A*, peaking at 24 h that resembled the situation in primary hPDLF [16]. Similarly, an increase in VEGF levels in gingival crevicular fluid of OTM patients was also detected after one day [7]. In contrast to in vitro models, levels of VEGF in patients remained high after seven days of treatment. The osteoblast marker *ALPL* showed a weaker induction than in primary hPDLF and the kinetics of its expression were faster. We also observed an induction of *M-CSF*, a cytokine controlling the differentiation of osteoclasts. This finding corresponds to reports demonstrating the induction of another osteoclastogenesis-inducing factor, *RANK-L*, upon mechanical loading [16] and is in line with reports showing increased M-CSF levels within gingival crevicular fluid during OTM [7]. 

Summing up, the majority of markers we had chosen as examples responded in PDL-hTERT cells in a manner similar to primary hPDLF. As a note of caution, we would like to emphasize that we selected only a few central markers for processes such as inflammation, osteoblastogenesis or osteoclastogenesis. Therefore, it remains to be established if further markers share the same behavior. The time course of the response to compressive force by the PDL-hTERT cell line might be slightly different to primary hPDLF, as we detected some more rapid responses (for ALPL) but also some slower ones (for PTGS2) than in primary hPDLF [16]. We would like to point out that the majority of examined genes follow very similar kinetics as seen in primary hPDLF [16], which may indicate an identical behavior.

To enable molecular gain- and loss-of-function studies, we searched for an efficient transfection protocol for PDL-hTERT cells. Transfection of a homogeneous cell population might strongly improve genetic manipulations such as overexpression or knockdown of a specific gene of interest. Indeed, we found an efficient method using Lipofectamine 2000. As PDL-hTERT cells are relatively sensitive to this transfection reagent, the amount has to be adjusted according to the exact experimental conditions. We achieved transfection efficiencies of ca. 10% at the cost of cell viability (ca. 40% survival rate). However, we still had reliable 6.60% transfection rates with much higher cell viability (ca. 75% survival rate) at a lower dose of Lipofectamine 2000.

In summary, due to a response to compressive force similar to primary hPDLF, the PDL-hTERT cell line has a good chance to facilitate basic research on periodontal mechanical stress by speeding up in vitro simulations. As a complement to established models using primary hPDLF, this cell line can reduce the need to harvest and cultivate primary cells from donors, which are not always readily available. What might even be a more important benefit is the possibility to transfect this homogeneous cell line, opening a vast array of molecular gain- and loss-of-function studies. 

## 4. Materials and Methods 

### 4.1. Cell Culture Experiments and In Vitro Model for Compressive Mechanical Strain

The PDL-hTERT cell line [23] was cultured in DMEM high glucose containing sodium pyruvate and GlutaMAX (Gibco, Thermo Fisher Scientific, Schwerte, Germany, 31966-021), 10% FCS (Bio&Sell, Feucht, Germany, S 0615) and 1% PenStrep (Gibco, 15140-122) at 37 °C and 5% CO_2_. Physiological compressive force was applied according to well-established protocols [3,16,19]. Cells were seeded in 6-well plates. When cells had reached a confluency of 70%, gravity-induced compressive force was applied by mechanical loading with sterile glass disks (33 mm Ø, 17.1 g, 2 g/cm^2^, Figure 1) for 6 h, 24 h, 48 h and 72 h. In parallel, control cells were grown on the same 6-well plates without compressive force. As reference, the starting point was used (T = 0 h). After the indicated time spans, medium was removed and stored at −80 °C until analysis and cells were used for extraction of RNA. Two independent experiments were performed (*n* = 2), each consisting of three wells with or without applied pressure, resulting in six independent samples (*n* = 6).

### 4.2. Isolation of RNA and Reverse Transcription

For extraction of RNA, the Qiagen RNeasy plus kit (Qiagen, Hilden, Germany, 74134) involving a step removing genomic DNA was used according to the manufacturer’s instructions. Concentration and purity of RNA was checked photometrically on a Implen NP80 nanophotometer. Absence of proteins was assured by OD260/280 values above 1.8. 1.2 µg of total RNA per sample were reverse transcribed with SCRIPT cDNA synthesis kit (Jena Bioscience, Jena, Germany, PCR-511S) using oligo-(dT)_20_ and random hexamer primers. First strand synthesis was performed at 42 °C for 10 min followed by 50 °C for 60 min. After heat inactivation (10 min at 70 °C), samples were immediately cooled on ice.

### 4.3. Quantitative Real-Time PCR

To assess cDNA amounts, quantitative real-time PCR was performed. Primers for qRT-PCR were used according to published literature; primer sequences are listed in Table 1. 

Quantitative real-time PCR was performed on a Roche LightCycler 96 with PowerUp SYBR Green Master Mix (Applied Biosystems, Thermo Fisher Scientific, Schwerte, Germany, A25777). Each PCR reaction was performed in technical triplicates, cDNA was pipetted as a 1:12 dilution. All other components were added as a master mix. PCR cycles were as follows: 5 min 95 °C, 40 cycles of 10 s 95 °C, 20 s annealing temp (annealing temperatures are listed in Table 1), 20 s 72 °C. Melting curves of PCR products were analyzed to assure their specificity. Relative gene expression was calculated by the ΔΔCt method with *PPIB* as reference gene [16,19].

### 4.4. ELISA

PGE2 secreted into cell supernatant was quantified by a commercially available enzyme-linked immunosorbent assay (ELISA) kit according to manufacturer’s instructions (Cayman Chemical, Biomol, Hamburg, Germany, 514010 (96)) with 1:50 dilution of supernatants. Optical density was measured at 450 nm on a BioTek Synergy H1 microplate reader with eight measurements per data point. Standard curve fit and calculation of concentrations were performed with a Cayman Chemical Excel Spreadsheet. 

### 4.5. Cell Transfection and Determination of Transfection Rate

Cells were grown under standard conditions to a confluency of 40% in 24 well plates. Transfections were performed in triplicate. For transfection with PEI, the following protocol was used: 1.5 µg of pEGFP-N1 plasmid was diluted in 150 µL DMEM (Gibco, 31966-021), 0.5 µL (low amount) or 1.5 µL (high amount) of PEI (Sigma-Aldrich, Merck, Darmstadt, Germany, 408727; diluted 1:2262, pH adjusted to 7.0) was added, immediately followed by a ten second mixing step on a vortex mixer. DNA-PEI complexes were allowed to form during a 15 min incubation period at room temperature. After that 50 µL were carefully dripped onto each well and the plate was cautiously shaken immediately. Cell culture medium was changed after 3 h. Transfection with SuperFect (Qiagen, 301305) was performed in the same way, except that 0.66 µL (low amount) or 2 µL (high amount) of SuperFect were used instead of PEI.

For transfection with Lipofectamine 2000 (Invitrogen, Thermo Fisher Scientific, Schwerte, Germany, 11668-027), 1.5 µg pEGFP-N1 were diluted in Opti-MEM medium (Gibco, 31966-021) to a final volume of 75 µL. In parallel, 0.33 µL (low amount) or 1 µL (high amount) of Lipofectamine 2000 were diluted in Opti-MEM to a final volume of 75 µL. Lipofectamine 2000 dilution was added to diluted DNA and carefully mixed. After a complex formation period of 5 min, 50 µL were carefully dripped onto each well and the plate was carefully shaken immediately. The next day, cell culture medium was changed.

After 48 h, cells were fixed with 4% formaldehyde for 10 min at room temperature, washed six times with PBS (Gibco™ 10010056) and permeabilized with 0.1% Triton X-100 (Carl Roth, Karlsruhe, Germany, 3051.3) in PBS for 10 min. Cell nuclei were visualized by incubation with 4′,6-Diamidin-2-phenylindol (DAPI; 1 mg/mL) at a 1:5000 dilution for 10 min. After washing with PBS, GFP-autofluorescence and DAPI were photographed on a Leica DMI6000 B inverted microscope equipped with a DFC 360FX camera. For estimation of cell toxicity of transfection mixtures, numbers of DAPI-positive nuclei per field of view were counted and set into relation to numbers of nuclei of non-transfected cells. For calculation of transfection efficiency, GFP-positive transfected cells were counted and set into relation to DAPI-positive nuclei of respective field of views.

### 4.6. Statistical Methods

For each analyte, the arithmetic mean of reference values at T = 0 h was set to 1. All values were calculated relative to this reference. Furthermore, values of samples with applied compressive force were divided by the arithmetic means of respective non-treated control samples. For statistical analyses, Kruskal-Wallis tests were performed in GraphPad Prism 8.3 (GraphPad Software, San Diego, California, USA) by comparing each mean with the mean of the reference (T = 0 h) with Dunn’s multiple comparisons test.

## Figures and Tables

**Table 1:** Primers used for qRT-PCR.

Target Gene	Forward Primer (5′-…-3′)	Reverse Primer (5′-…-3′)	Annealing Temperature	Reference
*ALPL*	ACAAGCACTCCCACTTCATCTG	GGTCCGTCACGTTGTTCCTG	60 °C	[16]
*COL1A2*	AGAAACACGTCTGGCTAGGAG	GCATGAAGGCAAGTTGGGTAG	60 °C	[16]
*FN1*	GCCAGTCCTACAACCAGTATTCTC	GCTTGTTCCTCTGGATTGGAAAG	60 °C	[16]
*IL-6*	TGGCAGAAAACAACCTGAACC	CCTCAAACTCCAAAAGACCAGTG	60 °C	[16]
*M-CSF*	GGAGACCTCGTGCCAAATTA	GGCATTGGGGGTGTTATCTC	60 °C	[30]
*P4HA1*	GCTCTCTGGCTATGAAAATCCTG	GTGCAAAGTCAAAATGGGGTTC	60 °C	[16]
*PPIB*	TTCCATCGTGTAATCAAGGACTTC	GCTCACCGTAGATGCTCTTTC	60 °C	[16]
*PTGS2*	GAGCAGGCAGATGAAATACCAGTC	TGTCACCATAGAGTGCTTCCAAC	60 °C	[16]
*VEGF-A*	TGCAGACCAAAGAAAGATAGAGC	ACGCTCCAGGACTTATACCG	58 °C	[16]

## Abbreviations

ELISA	enzyme-linked immunosorbent assay
hPDLF	human periodontal ligament fibroblasts
OTM	orthodontic tooth movement
PDL	periodontal ligament
PEI	Polyethylenimine
qRT-PCR	quantitative reverse transcriptase polymerase chain reaction
SEM	standard error of the mean
